# Vaginal Microbiome and Functional Pathway Alterations in Preterm Premature Rupture of Membranes Revealed by 16S rRNA Sequencing

**DOI:** 10.3390/life15101604

**Published:** 2025-10-15

**Authors:** Sangho Nam, Subeen Hong, In Yang Park, Sun Shin

**Affiliations:** 1 Department of Microbiology, College of Medicine, The Catholic University of Korea, Seoul 06591, Republic of Korea; 2 Department of Medical Sciences, Graduate School of The Catholic University of Korea, Seoul 06591, Republic of Korea; 3 Department Obstetrics and Gynecology, Seoul St. Mary’s Hospital, College of Medicine, The Catholic University of Korea, Seoul 06591, Republic of Korea

**Keywords:** preterm premature rupture of membranes, vaginal microbiome, 16S rRNA sequencing, maternal microbiome, vaginal dysbiosis

## Abstract

Preterm prelabor rupture of membranes (PPROM) is a leading cause of preterm birth and significant neonatal morbidity. The vaginal microbiome is implicated in its pathogenesis, but its detailed characteristics and functional consequences remain to be fully elucidated. This study aimed to provide a comprehensive, multi-faceted analysis of the vaginal microbiome and its functional potential in pregnant women with PPROM compared to healthy term controls. We collected vaginal fluid samples from eight PPROM and seven healthy control (HC) pregnant women. The vaginal microbiome was analyzed using 16S rRNA gene sequencing. We assessed community composition and state types (CSTs), alpha and beta diversity, co-occurrence networks, and predicted functional pathways using PICRUSt2. A molecular bacterial vaginosis (molBV) score was also calculated to determine the clinical relevance of the dysbiosis. The PPROM microbiome was characterized by a significant depletion of *Lactobacillus crispatus*–dominated communities (CST I) and a shift towards *L. iners*–dominated (CST III) or polymicrobial (CST IV) communities, which was consistent with a BV-positive molBV score. Alpha diversity was significantly higher in the PPROM group, and beta diversity analysis confirmed a distinct microbial structure between the two groups. Co-occurrence network analysis revealed a collapse of the protective, *Lactobacillus*-centered network in the PPROM group, which was replaced by a densely interconnected network of anaerobic bacteria with *Gardnerella vaginalis* as a key hub. Functionally, the PPROM microbiome was enriched for amino acid biosynthesis pathways, in contrast to the HC group, which was enriched for nucleotide and peptidoglycan biosynthesis. PPROM appears to be linked with a complex vaginal dysbiosis that encompasses significant alterations in microbial composition, diversity, interactions, and functional potential. These findings highlight the vaginal microbiome as a critical factor in the pathogenesis of PPROM and suggest its potential for risk stratification and as a therapeutic target to improve pregnancy outcomes.

## 1. Introduction

Preterm premature rupture of membranes (PPROM), defined as the rupture of fetal membranes before 37 weeks of gestation and prior to the onset of labor, accounts for approximately one-third of preterm births [1]. PPROM is associated with a markedly increased risk of serious prenatal complications, including chorioamnionitis, umbilical cord compression, placental abruption, neonatal sepsis, respiratory distress syndrome, and intraventricular hemorrhage, making it a leading cause of neonatal morbidity and mortality [2]. Despite its significant clinical burden, the pathophysiological mechanisms underlying PPROM remain incompletely understood.

*Lactobacillus*-dominated vaginal microbiota has long been considered a hallmark of health in the female reproductive tract. During pregnancy, the vaginal microbiome, particularly an environment dominated by *Lactobacillus* species, plays a critical role in maintaining reproductive tract homeostasis by maintaining a low pH environment to inhibit the growth of pathogens [3]. Conversely, a disruption of the *Lactobacillus*-dominant state, often accompanied by an overgrowth of anaerobic or bacterial vaginosis (BV)-associated taxa, has been linked to various adverse obstetric events [3,4,5].

The pathogenesis of PPROM is considered multifactorial, with intrauterine infection recognized as a major contributor that prematurely weakens the fetal membranes. In fact, positive amniotic fluid cultures have been reported in up to 36% of PPROM cases [6], suggesting that alterations in the vaginal microbiome may contribute to disease. Previous studies have shown that in PPROM, reduced *Lactobacillus* abundance and enrichment of BV-associated taxa, such as *Gardnerella vaginalis*, *Veillonella dispar*, and *Streptococcus mitis*, are associated with inflammation and the weakening of fetal membranes [7].

Despite these associations, recent studies on the relationship between spontaneous preterm birth and microbiome have often overlooked infectious factors, potentially introducing bias into their conclusions [8]. Moreover, most prior studies have focused solely on taxonomic composition without integrating analyses of microbial co-occurrence networks or functional capacities to uncover how specific microbial interactions and metabolic potentials might contribute to membrane rupture and the risk of preterm birth. Such comprehensive approaches are essential for elucidating how specific microbial interactions and metabolic potentials influence membrane rupture and preterm birth risk, revealing not only compositional shifts in microbial communities but also their potential functional consequences that may directly affect host–microbe interactions and pregnancy outcomes.

In this study, we conducted a comprehensive analysis of the vaginal microbiome in pregnant women with PPROM compared to healthy controls, integrating taxonomic profiling, alpha and beta diversity metrics, co-occurrence network analysis, and predictive functional pathway modeling. This multifaceted approach enables the identification of both compositional and functional alterations, providing novel insights into microbial community dynamics that may contribute to PPROM pathogenesis and adverse pregnancy outcomes.

## 2. Materials and Methods

### 2.1. Study Population

Fifteen pregnant women were recruited at Seoul St. Mary’s Hospital, the Catholic University of Korea, between October 2021 and October 2022. All participants were Korean, comprising eight women diagnosed with PPROM and seven healthy control (HC) women. The inclusion criteria were singleton pregnancy, maternal age between 19 and 50 years, and a gestational age of at least 18 weeks (Table 1). Women with a history of cervical cerclage during pregnancy were excluded. All participants received a detailed explanation of the study, and written informed consent was obtained prior to enrollment. The study was approved by the Institutional Review Board (IRB) of Seoul St. Mary’s Hospital (KC21TISI0621, approval date 1 November 2021) and conducted in accordance with the Declaration of Helsinki.

### 2.2. Vaginal Sampling

Vaginal swab specimens were collected by gently rubbing the posterior fornix of the vaginal wall using a flocked swab (NFS-2 swab, Noble Biosciences, Inc., Hwaseong-si, Republic of Korea) and suspended in 1 mL of phosphate-buffered saline (PBS). All specimens were collected prior to the administration of any antibiotic treatment. Samples were immediately transported to the laboratory and stored at −80 °C until further analysis.

### 2.3. Genomic DNA Extraction and Amplicon Sequencing

Genomic DNA was extracted from vaginal samples using the FastDNA Spin kit (MP Biomedicals, Irvine, CA, USA). The V3-V4 regions of the 16S rRNA gene were amplified using primers 341F (5′-AATGATACGGCGACCACCGAGATCTACAC-XXXXXXXX-TCGTCGGCAGCGTC-AGATGTGTATAAGAGACAG-CCTACGGGNGGCWGCAG-3′; underlined sequence indicates the target region primer) and 805R (5′-CAAGCAGAAGACGGCATACGAGAT-XXXXXXXX-GTCTCGTGGGCTCGG-AGATGTGTATAAGAGACAG-GACTACHVGGGTATCTAATCC-3′). PCR was performed using Herculase II Fusion DNA Polymerase (Agilent Technologies, Santa Clara, CA, USA) with the Nextera XT Index Kit v2 (Illumina, San Diego, CA, USA) under the following conditions: 95 °C for 3 min; 25 cycles of 95 °C for 30 s, 55 °C for 30 s, and 72 °C for 30 s; and a final extension at 72 °C for 5 min. Amplicons were verified by agarose gel electrophoresis, purified with CleanPCR (CleanNA, Waddinxveen, The Netherlands), and pooled in equimolar concentrations. Library quality was assessed on a 2100 Bioanalyzer (Agilent Technologies), and sequencing was performed on an Illumina MiSeq platform at CJ Bioscience, Inc. and Macrogen, Inc. (Seoul, Republic of Korea).

### 2.4. Bioinformatic Analysis

Raw sequence quality was assessed using FastQC (version 0.12.1) [9]. Primers and adapters were removed with Cutadapt (version 5.0) [10], and low-quality reads were trimmed using Trimmomatic (version 0.39) [11] with a sliding window (Q15, 4 bp). Reads shorter than 36 bp after trimming were discarded. Reads were merged with PANDAseq (version 2.11) [12] and filtered to remove sequences with a median quality score < Q20 or containing ambiguous bases.

Demultiplexed reads of the 16S rRNA V3-V4 region were analyzed using QIIME 2 (version 2024.02) [13]. Amplicon sequence variants (ASVs) were assigned taxonomy using a naïve-Bayes classifier trained on the 16S rRNA gene database (NCBI BioProjects 33175 and 33317), and assignments were validated by manual BLAST 2.15.0 search. ASVs present in fewer than two samples or with relative abundance <0.001% were excluded. Vaginal community state types (CSTs) were determined using VALENCIA [14], classifying samples into five CST groups: CST I (*L. crispatus*-dominated), CST II (*L. gasseri*-dominated), CST III (*L. iners*-dominated), CST IV (polymicrobial community with depleted lactobacilli), and CST V (*L. jensenii*-dominated). The VALENCIA score was computed on a scale from 0 to 1. In accordance with the established threshold, samples exhibiting a score below 0.5 were reassigned to a different CST to ensure reliable classification. We calculated the molBV index from 0 to 10, with larger values denoting increased microbial dysbiosis, using the R package molBV (version 1.0) [15].

Taxonomic features with relative abundance >0.001% in at least two samples were retained. Alpha diversity (Shannon, Simpson, Pielou’s evenness) was calculated using the R package vegan (version 2.6–6.1) [16]. Beta diversity was assessed in QIIME 2 plugin using unweighted UniFrac distance, PERMANOVA with 999 permutations, and principal coordinate analysis (PCoA) based on Jaccard distance.

Co-occurrence networks were constructed using the R package NetCoMi (network construction and comparison for microbiome data) (version 1.2.0) [17] based on Spearman correlations, and node sizes were scaled by Eigenvector centrality. Network plots were visualized using the R package igraph [18] and ggraph [19]. Functional pathways were predicted using PICRUSt2 (version 2.5.2) [20], and differentially abundant pathways between HC and PPROM were identified using MaAsLin2 (version 1.18.0) [21]. Only pathways with >5% contribution from a single species across all samples and adjusted *p* < 0.1 were retained. Also, functional pathway bubble plots were visualized using the R package ggplot2 [22].

### 2.5. Statistical Analysis

Comparisons between HC and PPROM groups were performed using the Wilcoxon Rank-Sum test, with *p*-values < 0.05 considered statistically significant. Relationships among the genera *Lactobacillus*, *Gardnerella*, *Streptococcus*, *Weissella*, and *Veillonella* were examined using Spearman’s rank correlation, with data normality assessed by the Shapiro–Wilk test. Correlation coefficients were calculated using the cor.test and function in the R package ggpubr (version 0.6.0) [23].

## 3. Results

### 3.1. Clinical Characteristics of Study Participants

A total of 15 pregnant women were enrolled in this study: eight with preterm prelabor rupture of membranes (PPROM) and seven healthy controls (HC). The demographic and clinical characteristics of the study groups are summarized in Table 1. No significant differences were found in maternal age and pre-pregnancy BMI between the groups (*p* = 0.378 and 0.444, respectively). However, the gestational age at delivery was significantly lower in the PPROM group compared to the HC group (34.10 ± 2.38 weeks vs. 38.44 ± 1.00 weeks; *p* < 0.001). The PPROM group also showed a trend towards a shorter cervical length (2.45 ± 0.79 cm vs. 3.34 ± 0.52 cm, *p* = 0.066) and lower 1 min and 5 min Apgar scores (median 6.5 vs. 9 and 8.5 vs. 10, respectively), although these differences did not reach statistical significance (*p* = 0.114 and 0.093, respectively). Correspondingly, 50% of infants in the PPROM group presented with a moderate (4–6) 1 min Apgar score, compared to only 14% in the HC group.

### 3.2. Hierarchical Clustering Analysis of the Vaginal Samples for HC and PPROM Groups

We obtained 272,629 high-quality sequence reads from 15 samples, with an average of 18,715 reads per sample (range: 1731 to 74,154). They were clustered into 122 unique amplicon sequence variants (ASVs), and all of which were successfully assigned a taxonomic classification (Supplementary Table S1).

**Figure 1:**
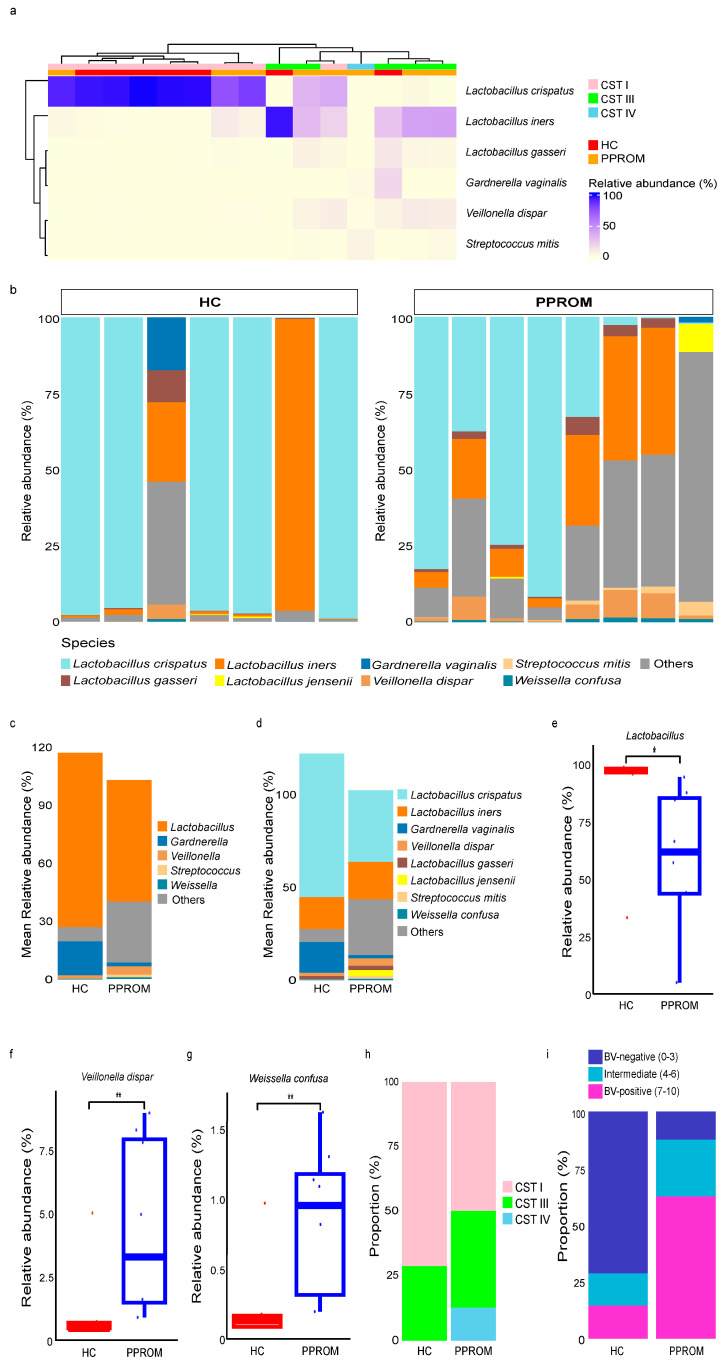
Relative abundance of vaginal microbiome composition by health status. (**a**) Heatmap showing the relative abundance of the top six bacterial taxa in the vaginal microbiomes of healthy control (HC) and women with preterm premature rupture of membranes (PPROM). Sample clusters with similar microbial compositions are indicated at the top of the heatmap, and taxa with similar abundance patterns are indicated on the left. All three CSTs were identified at the top of the clinical groups. CST I (*L. crispatus*–dominated; *n* = 9), CST III (*L. iners*–dominated; *n* = 5), and CST IV (a polymicrobial community with depleted lactobacilli; *n* = 1). (**b**) Bar plot showing the taxonomic composition and relative abundance of vaginal microbiota at the species level in HC and PPROM groups. (**c**,**d**) Stacked bar plots showing the mean relative abundance of vaginal microbiota at the (**c**) genus and (**d**) species level in the HC and PPROM groups. Each bar represents the average community composition for each group. (**e**) Relative abundance of *Lactobacillus* in the HC and PPROM groups. (**f**,**g**) The relative abundance of *Weissella confusa* and *Veillonella dispar* in the HC and PPROM groups. (**h**) Distribution of CSTs in the HC and PPROM groups. (**i**) Bar plot displaying the proportion of HC and PPROM groups based on molBV scores (BV-negative, intermediate, BV-positive). Statistical significance was indicated as follows: *p* < 0.05 (*), *p* < 0.01 (**).

Hierarchical clustering analysis partitioned the samples into two primary branches, which strongly corresponded with the clinical groups (Figure 1a). The first branch was predominantly composed of samples from the HC group and was characterized by the dominance of *Lactobacillus crispatus*. In contrast, the second, more heterogeneous branch contained all samples from the PPROM group, was defined by communities dominated by *Lactobacillus iners* or a polymicrobial state. At the individual sample level, the majority of samples in the HC group were characterized by a high dominance of *Lactobacillus* species, while the PPROM group exhibited greater heterogeneity with a decreased proportion of *Lactobacillus* (Figure 1b–d).

A Wilcoxon rank-sum test showed that the relative abundance of the *Lactobacillus* genus was significantly lower in the PPROM group compared to the HC group (*p* = 0.014) (Figure 1e). Conversely, species associated with dysbiosis were significantly elevated in the PPROM group, including *Weissella confusa* (*p* = 0.006) and *Veillonella dispar* (*p* = 0.009) (Figure 1f,g).

### 3.3. Community State Types (CSTs) and Correlation with Dysbiosis

To categorize the overall community structures, we classified the samples into CSTs. Three CSTs were identified in our cohort: CST I (*L. crispatus*–dominated; *n* = 9), CST III (*L. iners*–dominated; *n* = 5), and CST IV (a polymicrobial; *n* = 1). The distribution of these CSTs aligned with the clinical groups; the HC group consisted predominantly of CST I (5/7, 71.4%) with a few samples belonging to CST III, whereas the PPROM group exhibited a more diverse composition, comprising CST I (4/8, 50.0%), CST III (3/8, 37.5%), and CST IV (1/8, 12.5%) (Figure 1a,h, Supplementary Table S2).

To further quantify the degree of dysbiosis, we applied a molBV score based on the abundance of key anaerobic bacteria. The distribution of scores differed starkly between the groups (Figure 1i). The HC group consisted predominantly of BV-negative scores (71.42%, *n* = 5), whereas the PPROM group was dominated by BV-positive scores (62.5%, *n* = 5), indicating a significant shift towards a dysbiotic, BV-positive state.

### 3.4. Alpha and Beta Diversity

**Figure 2:**
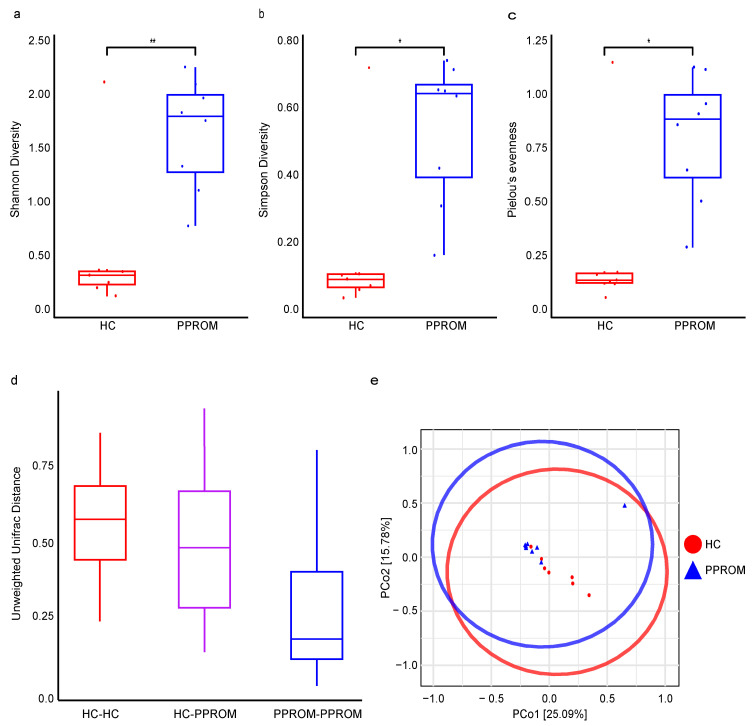
Different vaginal microbial alpha and beta diversity between the HC and PPROM groups. (**a**–**c**) Microbial alpha-diversity based on the Shannon, Simpson, and Pielou’s evenness index in the samples indicates a significant difference (* *p* < 0.05 and ** *p* < 0.01, respectively) between the groups based on the Wilcoxon Rank-Sum test (Mann–Whitney U test). The boxes represent the distributions of the alpha diversity index and show the median for each condition and 1.5 times the interquartile range (IQR). Whiskers extend to the furthest data point. (**d**) Box plot showing the distribution of unweighted UniFrac distance, indicating significant difference from each group, where a diversity distance of 0 represents identical bacterial composition of the vaginal microbiome and a distance of 1 indicates total dissimilarity. (**e**) Two—dimensional principal-coordinate analysis (2D-PCoA) of vaginal microbiota with different microbiota compositions based on Jaccard distance between the two groups (PERMANOVA analysis). The first two components of the variance are represented by Plotting HC (Red) vs. PPROM (Blue) samples with significant separation between the two groups. Each point corresponds to an individual sample. For each clinical group, an ellipse around the centroid is depicted. The sum of the first two components represents 40.87% of explanatory power, individually depicted in parentheses next to Axis1 and Axis2.

To compare the internal diversity of the vaginal microbial communities, alpha diversity was evaluated using Shannon, Simpson, and Pielou’s evenness indices at the species level. All three indices of alpha diversity were significantly higher in the PPROM group than in the HC group: Shannon diversity (*p* = 0.009), Simpson diversity (*p* = 0.013), and Pielou’s evenness (*p* = 0.020) (Figure 2a–c). These results indicate that the vaginal microbiome in the PPROM group is characterized by a greater number of species and a more even distribution compared to the less diverse, *Lactobacillus*-dominated communities of the HC group.

Beta diversity analysis also revealed a significant difference in the overall community structure between the HC and PPROM groups based on the unweighted UniFrac distance (PERMANOVA, pseudo-F = 2.31, *p* = 0.005) (Figure 2d). Principal Coordinates Analysis (PCoA) based on the Jaccard distance metric showed clearly distinct clusters of the HC and PPROM groups, which were statistically significant (PERMANOVA, pseudo-F = 1.62, *p* = 0.034) (Figure 2e). The first two principal coordinates (PCo1 and PCo2) explained a combined 40.87% of the total variance.

### 3.5. Co-Occurrence and Correlation Within the Vaginal Microbiome

**Figure 3:**
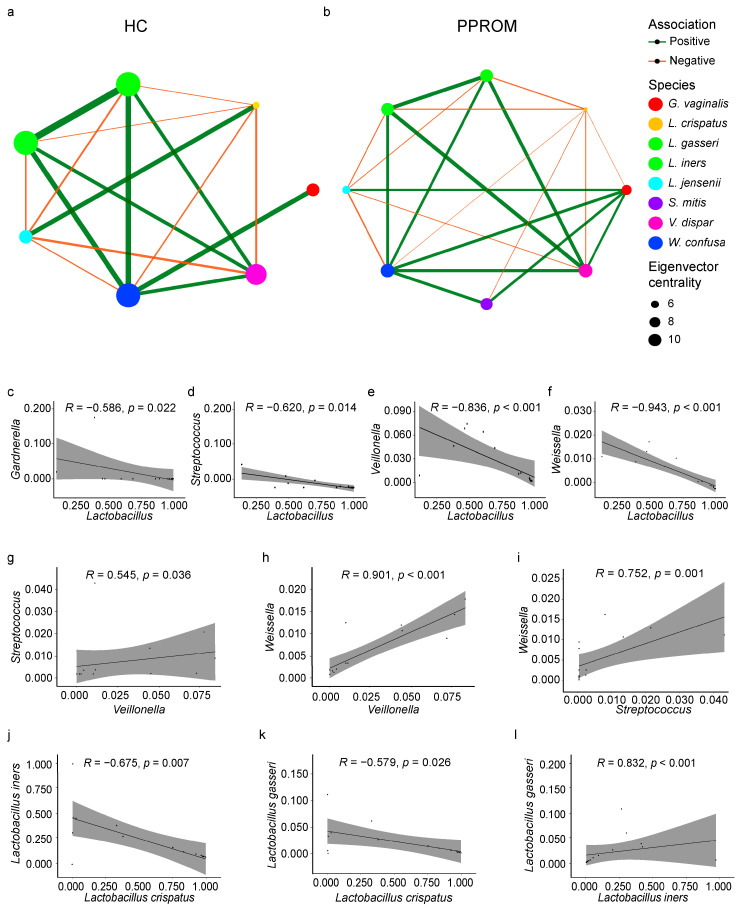
Network analysis identifying the correlation between HC and PPROM. Green-colored edges correspond to positive associations, and orange-colored edges represent negative associations. Node size denotes using Eigenvector centrality. (**a**,**b**) Correlation network of vaginal microbial taxa in HC and PPROM. Spearman (R) correlation plots showing the general theoretical relationship at the genus and species level. (**c**–**f**) Plots showing a negative correlation between the *Lactobacillus* genus, BV-associated, and non-*Lactobacillus* taxa. (**g**–**i**) Plots showing positive correlation among BV-associated and non-*Lactobacillus* taxa. (**j**–**l**) Plots showing both positive and negative correlation within *Lactobacillus* species. The strength and direction of the correlation are related to the magnitude and sign of the coefficient value R: strong correlations between ±0.50 and ±1. Statistically significant relationships, *p* < 0.05.

Co-occurrence network analysis revealed starkly different community structures between the clinical groups (Figure 3a,b). In the HC group, the network was characterized by positive correlations among beneficial lactobacilli (*L. crispatus* and *L. jensenii*), which were negatively correlated with anaerobic genera. The microbial community of the HC group formed a well-coordinated network, characterized by strong connectivity among similar nodes and distinct community boundaries (Supplementary Figure S1). In contrast, the PPROM network showed a collapse of this beneficial structure and the emergence of a new, tightly interconnected network among various anaerobic genera, with *Gardnerella vaginalis* acting as a key hub. The PPROM network exhibited increased dispersion and greater community heterogeneity (Supplementary Figure S2).

To further investigate these associations, we performed Spearman correlation analyses across all samples. The genus *Lactobacillus* showed significant negative correlations with dysbiosis-associated bacteria, including *Gardnerella* (R = −0.586, *p* = 0.0218), *Streptococcus* (R = −0.62, *p* = 0.014), *Veillonella* (R = −0.836, *p* < 0.001), and *Weissella* (R = −0.943, *p* < 0.001) (Figure 3c–f). Conversely, strong positive correlations were observed among these non-*Lactobacillus* BV-associated bacteria: for instance, *Veillonella* was positively correlated with both *Streptococcus* (R = 0.545, *p* = 0.036) and *Weissella* (R = 0.901, *p* < 0.001) (Figure 3g–i). Interestingly, the relationships within the *Lactobacillus* genus itself were also complex: *L. crispatus* abundance was negatively correlated with both *L. iners* (R = −0.675, *p* = 0.007) and *L. gasseri* (R = −0.579, *p* = 0.026), while *L. iners* and *L. gasseri* showed a strong positive correlation with each other (R = 0.832, *p* < 0.001) (Figure 3j–l).

### 3.6. Distinct Functional Pathways Between HC and PPROM Groups

**Figure 4:**
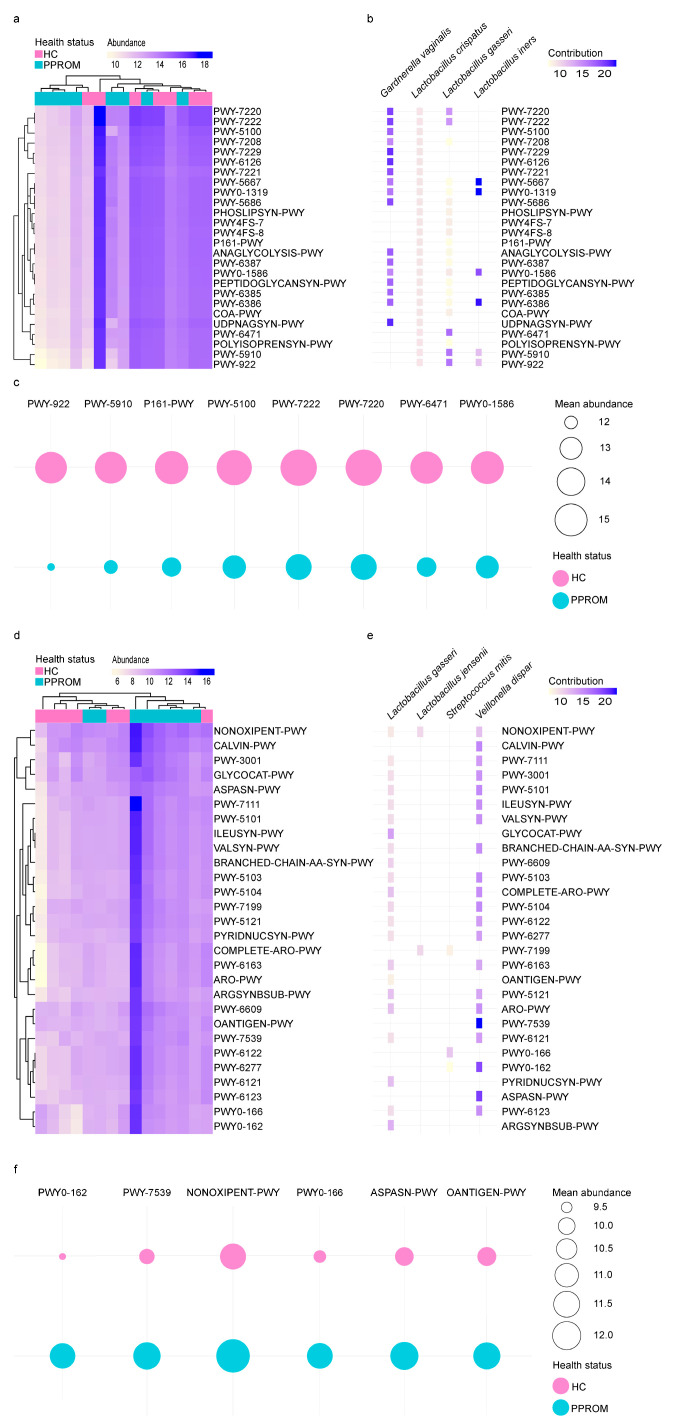
**Heatmap of the abundance of functional pathways and contributing species according to HC and PPROM groups.** Clusters of samples with similar functional properties emerging from the data are highlighted on top of the heatmap. Functional pathways that cluster due to similar patterns in the data are highlighted on the left side of the heatmap. Relative abundance data were log2-normalized for better visualization. Heatmap showing the abundance of 26 functional pathways that were significantly differentially abundant between women with HC and PPROM (**a**,**d**). The major contributing taxa for each functional pathway with significantly higher abundance in women with HC and PPROM (**b**,**e**). Bacteria that contribute to more than 5% of the total abundance in the cohort for each pathway were included in the visualization. (**c**–**f**) Bubble plots showing the mean abundances of significantly enriched pathways in each group. Eight pathways showed higher mean abundance in the HC, while six pathways were significantly enriched in the PPROM.

To investigate the metabolic potential of the microbial communities in our cohort, we performed a functional pathway analysis using PICRUSt2. Differential abundance analysis revealed significant shifts in the functional profiles between the groups. A total of 26 pathways, primarily related to nucleotide and peptidoglycan biosynthesis, were significantly enriched in the HC group (Figure 4a), and these functions were predominantly driven by *Lactobacillus* species, particularly *L. crispatus* and *L. iners* (Figure 4b). For instance, nucleotide biosynthesis (e.g., PWY-7220, PWY-7222) and pyruvate fermentation (PWY-5100) were largely attributed to *L. crispatus* and *L. iners*. Other species also made specific contributions; *L. gasseri* was a key contributor to certain peptidoglycan biosynthesis pathways (PWY-6471) and the mevalonate pathway (PWY-5910, PWY-922), while *G. vaginalis* contributed to another peptidoglycan pathway (PWY0-1586) and acetylene degradation (P161-PWY) (Figure 4c).

In contrast, 28 pathways were enriched in the PPROM group, many of which were associated with the biosynthesis of amino acids, such as L-isoleucine (Figure 4d). These pathways were driven by a more diverse set of bacteria, including *L. gasseri*, *L. jensenii*, *S. mitis*, and *V. dispar* (Figure 4e). *V. dispar* and *L. gasseri* were identified as the most significant contributors overall. Key functional contributions included the pentose phosphate pathway (NONOXIPENT-PWY) driven by multiple taxa, and L-lactate/heterolactic fermentation (PWY0-162, PWY0-166) driven by *S. mitis* and *V. dispar*. Furthermore, *V. dispar* was a major contributor to diphosphate biosynthesis (PWY-7539) and L-asparagine biosynthesis (ASPASN-PWY), while *L. gasseri* was the main contributor to O-antigen building blocks biosynthesis (OANTIGEN-PWY) (Figure 4f).

## 4. Discussion

In this study, we aimed to provide a comprehensive characterization of the vaginal microbiome in pregnant women with PPROM compared to healthy controls. Our findings reveal a profound shift towards dysbiosis in the vaginal microbial ecosystem of women with PPROM, characterized by a depletion of *L. crispatus*, an increase in microbial diversity, a fundamental reorganization of microbial interaction networks, and a distinct shift in the community’s metabolic potential.

The loss of *L. crispatus*, which is widely considered protective due to its high production of lactic acid, likely compromises the vaginal defense mechanisms against ascending pathogens [24]. This observation aligns with a growing body of evidence linking CST IV and bacterial vaginosis (BV) to adverse pregnancy outcomes, including preterm birth and associated adverse neonatal outcomes [3,25]. This dysbiotic process is often preceded by a shift towards *L. iners*, a transitional species whose unique metabolic profile—including the expression of the cholesterol-dependent cytolysin inerolysin—creates a less stable environment permissive to the expansion of BV-associated anaerobes [26,27]. These anaerobes contribute to pathology by producing enzymes like sialidases and proteases that degrade physical barriers, which in turn trigger inflammatory cascades involving matrix metalloproteinases such as MMP-8 and MMP-9, that culminate in PPROM [28,29].

Our findings are further corroborated by a recent meta-analysis, which demonstrated that PPROM is consistently associated with a significant reduction in protective species, such as *Lactobacillus* spp., and a concurrent dominance of a polymicrobial community including *G. vaginalis*, *Streptococcus* spp., and various opportunistic pathogens [30]. Furthermore, the strong correlation between PPROM status and molBV in our cohort further solidifies this link, suggesting that the observed dysbiosis is not only statistically significant but also clinically relevant in a more precise, molecularly defined context.

In our hierarchical clustering analysis, the grouping of *L. iners*-dominated samples (CST III) with the dysbiotic polymicrobial communities, rather than with the stable *L. crispatus* communities, highlights the complex and species-specific roles that *Lactobacillus* plays in defining the vaginal environment. While also a *Lactobacillus*-dominated community, CST III is often considered less stable and more transitional than the robustly protective CST I. This is attributed to the unique characteristics of *L. iners*; its smaller genome and the production of the inerolysin allow it to adapt and persist in the less acidic and more diverse environments characteristic of impending dysbiosis [27,31,32]. Therefore, a shift from an *L. crispatus*- to an *L. iners*-dominated microbiome may represent a critical step towards an unstable ecosystem, one more susceptible to the establishment of the polymicrobial networks observed in our PPROM group.

Beyond simple compositional changes, our network analysis revealed a fundamental reorganization of microbial interactions. In the HC group, the network was structured around beneficial lactobacilli acting as a stable core, antagonizing anaerobic species. In contrast, this protective structure collapsed in the PPROM group and was replaced by a new, densely interconnected network of anaerobic bacteria. The emergence of *G. vaginalis* as a central hub in the PPROM network may facilitate a cooperative environment that supports the growth and pathogenic activity of other anaerobes, thereby amplifying the dysbiotic state and its inflammatory consequences [33,34].

The observed microbiome shifts were accompanied by significant alterations in the predicted metabolic potential of the microbiome. Increased proteolysis and amino acid metabolism by anaerobic bacteria can produce pro-inflammatory compounds such as amines and certain short-chain fatty acids, which are known to be associated with BV and may contribute to cervical weakening and local inflammation [35,36,37]. Notably, *V. dispar*, a species significantly elevated in the PPROM group, was predicted to be a major contributor to several of these dysbiotic pathways, including L-lactate fermentation and L-asparagine biosynthesis. The prominence of *V. dispar*, a bacterium commonly found in the oral and gut microbiomes, may also suggest a potential translocation from distant microbial sites, warranting further investigation into an oral-vaginal axis [38]. Functionally, the enrichment of amino acid biosynthesis pathways in the PPROM group is concerning, as their metabolic byproducts (e.g., amines) can exacerbate the inflammatory cascade that directly impacts the fetus. This contrasts with the healthy state, where the microbiome’s function is geared towards maintaining a stable, protective environment for the developing fetus.

By combining microbial interaction networks and predicted metabolic functions, this study provides a comprehensive, multi-faceted view of the PPROM microbiome that moves beyond prior studies focused solely on taxonomic composition; however, its findings should be considered in light of certain limitations inherent to its exploratory design. Our preliminary conclusions are drawn from a small cohort (*n* = 15), which, while revealing clear trends, requires validation in larger prospective studies to confirm generalizability. The cross-sectional nature of our design powerfully demonstrates a strong association between dysbiosis and PPROM, but longitudinal studies are needed to establish a causal relationship. Future prospective cohort studies that track the vaginal microbiome from earlier gestation are therefore essential to identify predictive biomarkers and pinpoint critical windows for potential intervention. Additionally, although we sampled within a defined gestational window (32–35 weeks), the PPROM samples were collected at a relatively earlier gestational age within this range, which might have influenced the results. To overcome the limitations of 16S rRNA sequencing, a shotgun metagenomics approach would provide higher-resolution results and help identify functional genes. The discovery of bioactive metabolites through a metabolomics approach, combined with genomics, creates a multi-omics approach that will be vital for developing robust biomarkers for early risk stratification and designing targeted microbiological interventions.

In conclusion, our integrated analysis reveals a key link between PPROM and a dysbiotic vaginal microbiome, marked by shifts in composition, diversity, network structure, and metabolic potential. This multifaceted perspective supports a model where vaginal dysbiosis is not merely a coincident finding but may be an active contributor to the pathophysiology of PPROM.

## Figures and Tables

**Table 1:** Clinical characteristics of the study subjects.

	PPROM (*n* = 8)	HC (*n* = 7)	*p*-Value
Maternal age, years (mean ± SD)	36.75 ± 1.75	34.57 ± 4.04	0.378
Pre-pregnancy BMI (kg/m^2^)	36.16 ± 6.34	32.50 ± 3.35	0.444
Gestational age at sampling, weeks (mean ± SD)	34.39 ± 2.52	36.56 ± 0.87	0.022
Gestational age at delivery, weeks (mean ± SD)	34.10 ± 2.38	38.44 ± 1.00	3.11 × 10^−4^
Cervical length, cm (mean ± SD)	2.45 ± 0.79	3.34 ± 0.52	0.066
1 min Apgar score (median (Q1–Q3))	6.5 (5–9)	9 (8–9)	0.114
Moderate (4–6), *n* (%)	4 (50%)	1 (14%)	
Vigorous (7–10), *n* (%)	4 (50%)	6 (86%)	
5 min Apgar score (median (Q1–Q3))	8.5 (6.5–10)	10 (10–10)	0.093
Moderate (4–6), *n* (%)	2 (25%)	0 (0%)	
Vigorous (7–10), *n* (%)	6 (75%)	7 (100%)	

Two-tailed Mann–Whitney U test. BMI, body mass index.

## Data Availability

The original contributions presented in this study are included in the article/Supplementary Materials. Further inquiries can be directed to the corresponding author.

## Supplementary Materials

The following supporting information can be downloaded at: https://www.mdpi.com/article/10.3390/life15101604/s1. Table S1: Abundance and taxonomy of vaginal microbial ASV in the HC and PPROM groups; Table S2: VALENCIA score; Figure S1: Network correlation matrix in the HC group; Figure S2: Network correlation matrix in the PPROM group.
