# Supplementary figures and images for: Vaginal Microbiome and Functional Pathway Alterations in Preterm Premature Rupture of Membranes Revealed by 16S rRNA Sequencing
Source: Life (Basel). 2025 Oct 15;15(10):1604. doi: 10.3390/life15101604 (PMC12565210; doi:10.3390/life15101604)

Figure S1. Correlation matrix about HC group.

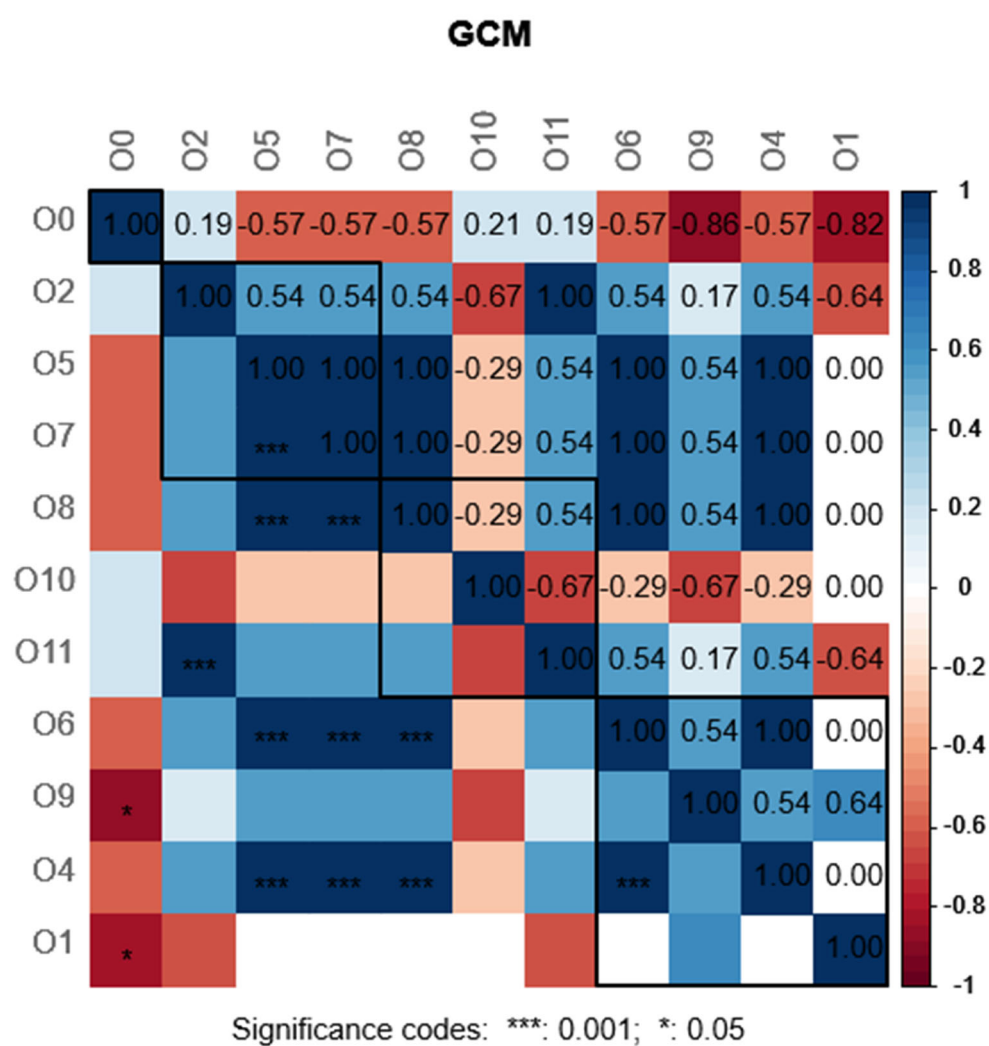

Figure S2. Correlation matrix about PPRM group.

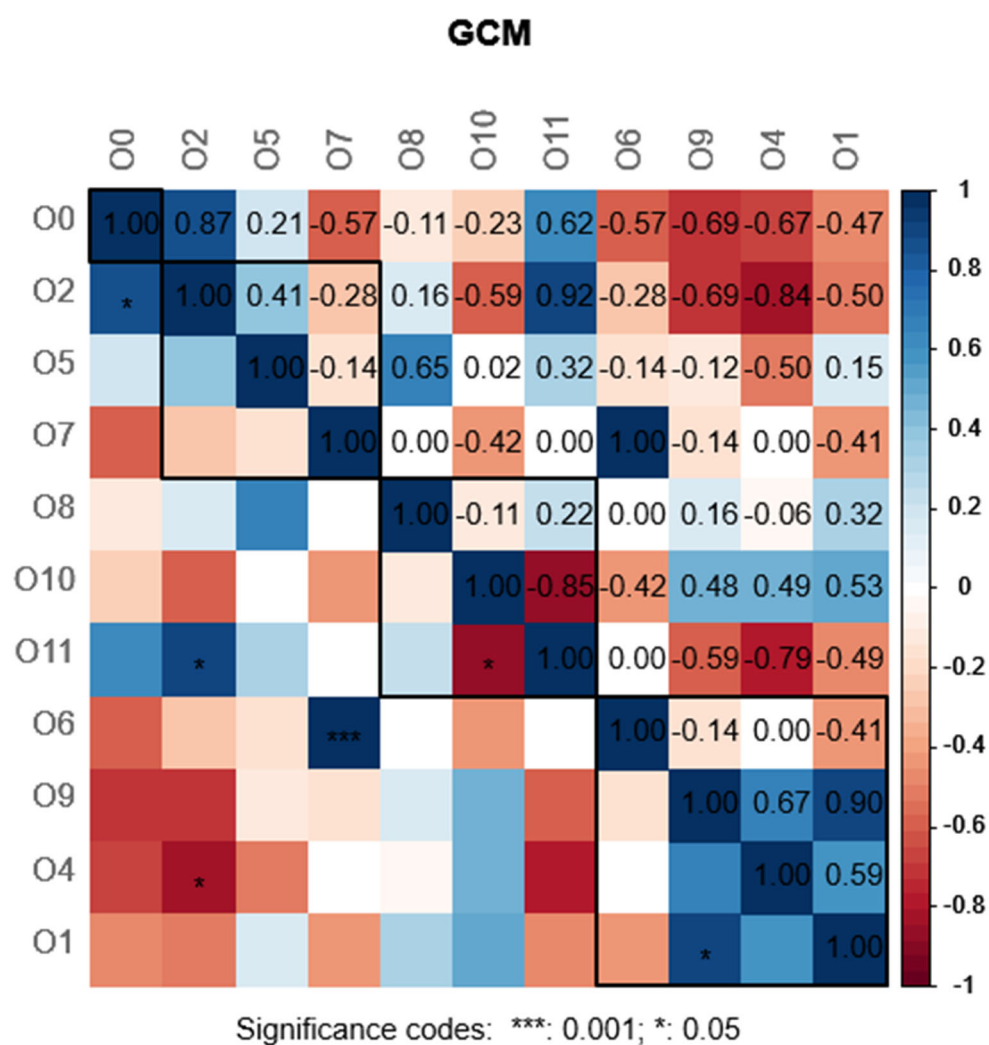

Supplement: Supplementary file 1 [file life-15-01604-s001.zip › life-3911321-supplementary.pdf]
